# Aging and Work Ability: The Moderating Role of Job and Personal Resources

**DOI:** 10.3389/fpsyg.2017.02262

**Published:** 2018-01-10

**Authors:** Daniela Converso, Ilaria Sottimano, Gloria Guidetti, Barbara Loera, Michela Cortini, Sara Viotti

**Affiliations:** ^1^Department of Psychology, Università degli Studi di Torino, Turin, Italy; ^2^Department of Psychological Sciences, Università degli Studi “G. d’Annunzio” Chieti – Pescara, Chieti, Italy

**Keywords:** aging workers, work ability, job resources, personal resources, nurses

## Abstract

**Objective:** Demographic changes involving western countries and later retirements due to the recent pension reforms induce a gradual aging of the workforce. This imply an increasing number of workers with health problems and a decreasing of ability to work. In this direction, the present study aims at examining the role of job and personal resources between age and work ability within nurses.

**Method:** The study was cross-sectional and not randomized; data were collected by a self-report questionnaire during a multi-center survey conducted in two Italian hospitals in 2016. In this way, 333 nurses were reached.

**Results:** Multiple linear regression showed that age is significantly and negatively associated to work ability, and that job resources (e.g., decision authority and meaning of work) and personal resources (e.g., hope and resilience) moderate the relationship between age and work ability.

**Discussion:** These results highlight that investing in work and personal resources to support WA is even more relevant for those professions where high physical effort is required.

## Introduction

Aging is mainly considered from an individual perspective as the process involving any human being throughout his or her lifespan. Alternatively, it can be considered a global phenomenon that is now affecting entire countries and populations: processes and changes must be considered in this “new” perspective (new: for the first time in history, older people have become the largest part of the population) not as single trajectories but in a systemic manner, taking into account the individual and the social changes as well as their interactions.

It is renowned that western countries (and in the near future, many others in the east and in the south) are getting older: the decline of fertility and low birth rates on one hand and rising life expectancies on the other are determining the increase in the mean age of the population. The higher mean age and growing of the “old-old” cohort (+80) imply problems for the sustainability of the welfare and the pension systems. Later retirements due to the consequent reforms adopted and the aforementioned demographic changes induce in turn a gradual aging of the workforce: in Europe the employment rate in the 55–64 years’ cohort increased 9.8% points (Eurostat, UE28, 2008–2016); the percentage of workers over 55 in Italy was 46.2% in 2014 (Eurostat, 2016).

The aging of workers requires attention on two major issues, that in the sustainability perspective (Miglioretti et al., 2016; Gragnano et al., 2017) are kept together: the first one involves workers’ health, the second one job productivity and performance.

A larger number of older workers implies, for example, an increasing number of people at work with minor and major health problems that occur more frequently after 55 years of age (Knoche et al., 2012; Maricchio et al., 2013). In this view, over the next several years organizations will be faced with a prevalence rate of chronic diseases (e.g., musculoskeletal disorders, diabetes, or cardiovascular diseases, cancer) close to 20/30% of the entire workforce (Gragnano et al., 2017), with an attendant strong impact on work ability (WA) (Camerino et al., 2006, 2008a; Golubic et al., 2009; Milosevic et al., 2011; Carel et al., 2012; Monteiro et al., 2012; Loera et al., 2013; Guglielmetti et al., 2014; Leijten et al., 2014; Converso et al., 2015b; Viotti et al., 2017a). Therefore, aging requires workers’ health and diseases to be reconsidered from the perspective of a “work-health balance” (WHB: Miglioretti et al., 2016; Gragnano et al., 2017) to promote interventions (age management policies, job design from an ergonomic perspective, job redesign addressing psychological changes: Truxillo and Zaniboni, 2017) aimed at supporting workers’ mental and physical health, WA, and job productivity over the entire working lifecycle, and over: higher-quality jobs (e.g., complex and challenging works) can impact cognitive development, reducing the risk of dementia and enhancing cognitive ability in later life (Andel et al., 2005; Finkel et al., 2009), and in general strengthening physical and psychological health during work-life is of great relevance even for late-life health and function (Nilsen et al., 2017).

### Work Ability (WA)

The Work Ability Index (WAI) is a widely used tool that measures the perception of sustainability at work derived from the construct of WA, developed by Ilmarinen and the research group of Finnish Institute of Occupational Health in the 1980s. In the past, this construct was used mainly by occupational physicians, but it is now fully integrated in the occupational health psychology (OHP) literature (Brady et al., 2016).

WA describes the physical and intellectual resources on which individuals can rely to respond to the emotional, cognitive, and physical demands posed by their work (Tuomi et al., 1991, 2001). WA has a strong health-related nature; hence, it inevitably decreases with age, even if there is a high rate of variability among individuals, as widely demonstrated by international research (Ilmarinen et al., 1997; Pojonen, 2001; Goedhard and Goedhard, 2005; Camerino et al., 2006; Lin et al., 2006; Monteiro et al., 2006; van den Berg et al., 2008, 2009; Converso et al., 2015b; Godinho et al., 2016) that highlights, for example, the same trend within different European countries (Camerino et al., 2006). Ilmarinen and Ilmarinen (2015) explained that this decline is due to the imbalance between the individual resources and the demands of the job, namely the work and the organizational context are not aligned to the age-related changes in personal resources. Poor WA is predictive of sickness absences, early retirement, disability pension, intention to leave (Goedhard and Goedhard, 2005; Camerino et al., 2006; Monteiro et al., 2006; Emberland and Knardahl, 2015), work stress, depression (Tuomi et al., 1991; van den Berg et al., 2008; Bethge et al., 2009; Godinho et al., 2016), and emotional exhaustion (Viotti et al., 2017a). Conversely, good WA is associated with a high quality of work, high productivity, and enjoyment of time at work. WA also predicts good quality of life, well-being, and active and meaningful retirement (Tuomi et al., 2001).

### WA in the Working Life Span

A recent review of the WA model, appropriate for an aging workforce process, the WA-PR (Work Ability Personal Radar), assumes a multidimensional view of WA based on the self-assessment of subjective experiences of personal resources, working context, and work–life interface and proposes the metaphor of a house, whose floors represent the four main dimensions of WA (Ilmarinen et al., 2005, 2015). Health and Functional Capacity is the first floor of the house and consists of physical, mental, and social resources; Occupational Competence, the second floor, consists of expertise, skills, and knowledge acquired through experience, years of work, training, and education; Attitude and Motivation, situated on the third floor, are the reasons people work and are influenced by previous work experiences; Working Conditions correspond to the fourth floor and consist of work and all of its dimensions. If the resources of the individual (floors 1–3) are in balance with the fourth floor, the WA is good. If the individual resources are in imbalance with the working conditions, WA is deteriorating. Organizational support is of great importance in relation to the fourth floor, while the personal resources are of great importance in relation to the first three floors.

Altogether, the floors of the WA house are in relation to a context of life that consists of work, family, and spare-time activities, representing the fifth floor (Ilmarinen et al., 2015).

This new multidimensional model of WA overcomes the previous conceptualization, mainly focused on an individual state, and coherently with other OHP models (Job-Demand-Control, JD-C, of Karasek, 1979 and Job-Demand-Resources, JD-R, of Demerouti et al., 2001) consider the interaction between personal resources (the first three floors) and the job demands and resources (the fourth floor). As it is assumed by the JD-R model (Demerouti et al., 2001), work factors which could affect workers’ psychological health can be classified into two categories: job demands and job resources. Job demands refer to those aspects of job that require the workers to sustain psychological or physical effort and are associated with cost in terms of health. Job resources refer to those physical, psychological, social, or organizational aspects of the job that may help to achieve work goals, reduce job demands and the related physiological and psychological costs, and stimulate personal growth and development.

The structure of WA changes during a person’s life and career as, for example, aging affects the individual’s resources or personal resources like competence and experience that may completely or partially compensate for physical “losses” and changes (Baltes and Baltes, 1990). Moreover, when people perceive their time as limited (as older workers do), emotional goals take priority and workers tend to focus their attention on affective rewards and resources (Carstensen et al., 1999; Truxillo et al., 2012) rather than on material or career-based rewards. As Demerouti et al. (2001) have affirmed, each work environment includes different demands and resources that interact with personal resources (Xanthopoulou et al., 2007) developing a dual process, the health impairment process and the motivational process, which may vary throughout the lifespan, when the same job demand may be perceived as more demanding, or the same resource may be perceived as less supportive. In other words, job well-being along the entire work lifespan is the result of an ever–changing balance between individual resources and job demands.

### WA in Relation to Job and Organizational Characteristics

Several studies have shown that WA is influenced by individual, job-related, and lifestyle factors (Tuomi et al., 1998; Camerino et al., 2006; Bethge et al., 2009; Matzolumi et al., 2012; McGonagle et al., 2014, 2015; Airila et al., 2014; Converso et al., 2015a; Godinho et al., 2016; Li et al., 2016; Sottimano et al., 2017; Viotti et al., 2017b). To understand the factors that sustain WA, researchers have analyzed specifically the role of job resources in supporting WA, both directly (Tuomi et al., 1991; Larsson et al., 2012; Matzolumi et al., 2012; Airila et al., 2014; McGonagle et al., 2014; Emberland and Knardahl, 2015; Li et al., 2016) and indirectly, by buffering the negative effects of job demands on WA (McGonagle et al., 2014; Viotti et al., 2017b). Specifically, studies have highlighted the direct effect of decision authority, skill discretion, social support, and the meaning of work on WA in various working populations (van den Berg et al., 2008, 2011; Weigl et al., 2013; McGonagle et al., 2014; Li et al., 2016; Sottimano et al., 2017). For example, Li et al. (2016) showed that social support was positively and directly associated with WA, while Weigl et al. (2013) and McGonagle et al. (2015) underlined the importance of control in maintaining WA. In particular, Weigl et al. (2013) have shown that job control moderates the negative impact of aging on WA. In this direction, in a study by McGonagle et al. (2014) among healthcare workers, supervisor support and skill discretion were found to moderate the negative relationship between job demands and WA. Schulz et al. (2017) have highlighted similar findings: team health climate emerged as particularly important for older employees’ WA because the social context may support those compensation strategies that older workers may employ when job demands exceed their physical or mental capabilities.

Furthermore, the lifespan perspective on work design (Truxillo et al., 2012; Zacher and Schmitt, 2016) proposes that some job resources are more positively related to indicators of occupational well-being among older workers. More specifically, the literature shows that decision authority (Zacher and Frese, 2009; Zacher et al., 2010; Truxillo et al., 2012; Zaniboni et al., 2016; Viotti et al., 2017b), skill discretion (Truxillo et al., 2012; Zaniboni et al., 2013, 2014; Viotti et al., 2017b), social support (Truxillo et al., 2012; Drabe et al., 2014; Li et al., 2016), and meaning of work (Sottimano et al., 2017; Viotti et al., 2017b) are very important dimensions to promote WA and, more generally, well-being at work, in particular within older workers.

### WA in Relation to Personal Resources

Personal resources are considered positive self-evaluations that are linked to resilience and refer to an individual’s sense of ability to successfully control or impact his or her environment (Hobfoll et al., 2003); they may have similar motivational potential to those of job resources (Xanthopoulou et al., 2007). For example, Youssef and Luthans (2007), in the psychological capital model perspective, found that hope had a positive effect on employee satisfaction and work happiness (Luthans, 2002; Luthans and Youssef, 2004); other researchers have shown that optimism correlates with employee engagement and performance (Medlin and Faulk, 2011), while resilience positively correlates with workplace performance and work happiness (Luthans et al., 2008).

Few studies have specifically considered the relationship between WA and personal resources. In this context, Airila et al. (2014) highlighted that personal resources predicted WA both directly and indirectly via work engagement, whereas the studies by Larsson et al. (2012), specifically conducted within nurses and nurse aides, and Ng et al. (2015) revealed that self-efficacy contributed to increased WA.

### WA in the “High Touch” Professions: When Physical and Emotional Demands Cannot Be Reduced

As previously stated, the topic WA changes across the lifespan and needs to be addressed differently according to the job characteristics, the job demands, and the organizational context. According to Warr (1994), four main types of work emerge from the interaction between age/experience and job demand: (a) the first one includes jobs in which the required skills do not decline with age and experience is an added value to the work; (b) in the second one, the skills required do not decline with age, but experience does not constitute an added value to the work; (c) the third type includes workers whose skills decline with age, but for which experience can partially compensate; (d) in the fourth, the required skills decline with age and experience cannot adequately compensate. In this direction, the literature shows that the relationship between age and ability to work is more critical when the workers are engaged in jobs with high physical demands (McGonagle et al., 2015). Often, for these professions, an early retirement is provided or a transition to less-physically demanding job tasks is made available within the same organization. In addition, WA can be supported through prevention activities and radical job redesign interventions: a famous example of good practice is the BMW intervention developed in 2007 (Loch et al., 2010), where a relatively low-cost project mainly directed toward preventing musculoskeletal disorders in a high-physically demanding job where the workforce was significantly aging, increased the productivity by 7%. Introducing in the first experiment 70 ergonomic changes, like installing special chairs at several workstations, allowed workers to spend the majority of their working hours sitting down or relaxing for short periods; vertically adjustable tables to adapt the workstations to each worker’s height, and so reducing back strain; special lenses to help workers distinguish among small parts, reducing eyestrain and mistakes due to the decline of sight. As scholars affirmed, BMW “defused the demographic time bomb” redesigning its factory for and with older workers, who were involved asking them to imagine how to ameliorate their working conditions in light of the aging process (Loch et al., 2010).

When the job demands are mainly cognitive, as in most of the type A or B jobs, WA decreases slowly and a large number of interventions devoted to sustainability in the organizational context can be developed. Regarding emotionally demanding jobs, they may be included in types A or C as, for example, job experience can be helpful in contrasting customer-related stress. One specific case is represented by jobs having both high physical and emotional demands, as in healthcare and pre-school contexts, where job redesign interventions or the adaptation of work environment are more complex or impossible to promote because of the specific nature of the work. Taking care of patients, babies, children, and sick people represent the core activity of these jobs that cannot be reduced or changed, and the continuous exposure to bio-mechanical risks may increase musculoskeletal disorders (Phongamwong and Deema, 2015), especially during aging, and negatively impact WA (Prochnow et al., 2013). As highlighted by previous studies conducted among preschool teachers aged 50 or over, the perception of poor WA (associated with physical problems) can mediate the relationship between demands/resources and worker well-being (Viotti et al., 2017a).

In light of the above, the present study aims then at examining the role of job and personal resources between age and WA within a typical *“high touch” profession:* nurses. We considered as *job resources* three dimensions on the basis of the previously described WA-PR house model and of the studies on the relationship between job resources and WA (Weigl et al., 2013; McGonagle et al., 2015; Li et al., 2016): decision authority that describes the autonomy in making job-related decisions (as autonomy), skill discretion (Viotti and Converso, 2016) that describes the opportunity to use certain skills at work (as competence), and social support (Li et al., 2016), as WA in the nursing context can be supported by job resources of an interpersonal nature, such as social support (Hägglund et al., 2011; Airila et al., 2014) expressed by colleagues, superior and patient as well (Converso et al., 2015a); meaning of work that can be considered as a type of intrinsic reward referred to job meaningfulness and constructiveness (Viotti et al., 2017b), for its importance in the field of the “helping professions.”

Concerning *personal resources*, we considered the following factors: self-efficacy, hope, optimism, and resilience. Self-efficacy refers to people’s beliefs in their capabilities to produce desired effects by their own actions (Bandura, 1977); hope refers to a positive motivational state; optimism refers to the tendency to expect positive events in one’s life; and resiliency refers to successfully coping with adversity or stress.

We hypothesize the following:

(H1) Age is negatively associated with WA.
(H2) Job and personal resources are positively associated with WA.
(H3) Job and personal resources moderate the negative relationship between age and WA. That is to say, the relationship between age and WA is stronger in conditions of low resources and weaker in conditions of high resources, both job and personal resources.

## Materials and Methods

### Data Collection and Participants

The study was cross-sectional and non-randomized. Data were collected by means of a self-report questionnaire during a multi-center survey conducted in two hospitals in a region of North–West Italy in 2016. Questionnaires were distributed during working hours. Workers were asked to enclose the completed questionnaire in an envelope and leave it in a box that the research team had placed in each unit/service. In total, 524 nurses were reached and 333 questionnaires were returned to the research team (response rate: 63.54%). The majority were women (female = 85.9%; male = 14.1%), with an age ranging from 23 to 64 years (*M* = 44.65, *SD* = 16.02). The average job seniority in the health sector was 19.53 years (*SD* = 10.61) and ranged from less than 1 to 42 years. Finally, 64% of the sample worked on the night shift.

The distribution of the socio-demographic data in this sample is analogous to the distribution in the Italian nursing workforce employed in the public health sector in which 65% are women and 35% are men, the average age is 44.6, and the average job seniority is 17 years^1^.

### Ethical Considerations

Hospital administrations evaluated, endorsed, and authorized the research, allowing researchers to use the data for scientific purposes. Upon approval, department chiefs and nursing coordinators from each unit/service were asked for authorization to administer the questionnaire to the nurses. An additional ethical approval was not required since there was no treatment, including medical, invasive diagnostics, or procedures, causing psychological or social discomfort for the participants, nor were patients the subject of data collection.

Participation in the survey was voluntary. The research conforms to the provisions of the Declaration of Helsinki in 1995 (as revised in Edinburgh 2000), and all ethical guidelines were followed as required for conducting human research, including adherence to the legal requirements of the study countries.

### Measures

The questionnaire was developed specifically for this study. It included items aimed at collecting age, other socio-demographic information (i.e., gender, job seniority, and night shift) and sub-scales aimed at measuring study variables (i.e., work ability, job resources, personal resources, and physical demands).

#### Job Resources

Four subscales from the Job Content Questionnaire (JCQ, Karasek, 1985) were employed to measure skill discretion (SD, five items, α = 0.83, e.g., “My job requires that I learn new things”), decision authority (DA, three items, α = 0.62, e.g., “My job allows me to make a lot of decisions on my own”), support from colleagues (SC, five items, α = 0.82; e.g., “People I work with are competent in doing their jobs”), and support from superiors (SS, four items, α = 0.85; e.g., “My supervisor is helpful in getting the job done”). Finally, meaning of work (WM, three items, α = 0.75, e.g., “My work is meaningful”) from the Copenhagen Psychosocial Questionnaire (COPSOQ, Kristensen et al., 2005) was considered. Responses on all sub-scales were given on a 4-point scale with a range from 1 = strongly disagree to 4 = strongly agree.

#### Personal Resources

Work-related self-efficacy was measured using a scale developed by Caprara (2001), consisting of five items (SE, e.g., “At work, I’m able to manage any emergency and deal with unexpected tasks,” α = 0.74). Hope (HO, e.g., “I’m grateful to my past experiences, which have prepared me well to succeed in the face of challenges,” α = 0.79), and optimism (OPT, e.g., “Even when facing work hardships, I expect things to turn out for the best,” α = 0.66), contained seven items each, and were developed by Snyder et al. (1996) and Carver et al. (2009), respectively. Resilience (RES, e.g., “At work, I am able to adapt to any change required by the situation,” α = 0.83) contained 10 items and was developed by Campbell-Sills and Stein (2007). Responses on these scales were given on a 4-point scale with a range from 1 = not true to 4 = completely true.

#### Outcome

Work ability was measured with the Work Ability Index (WAI, Tuomi et al., 1998), which contains seven sections, each of which gives a partial score that contributes to forming the overall WA score (ranging from 7 to 49). The seven sections measure: (1) current WA compared with lifetime best (range of the score: 1–10); (2) WA in relation to mental and physical demands (range of the score: 2–10); (3) number of current diseases diagnosed by a physician (range of the score: 1–7); (4) estimated work impairment due to diseases (range of the scores 1–6); (5) sick leave during the past 12 months (range of the scores: 1–5); (6) self-prognosis of WA for the next 2 years (scores: 1–4 or 7); and (7) mental resources (range of the score: 1–4). Cronbach α is equal to 0.73.

#### Control Variables

Gender, job seniority, and night shift were included as control variables in the models as it is recognized that they may represent potential confounders in the relationship between age and WA (Ilmarinen et al., 1997; Camerino et al., 2008b).

### Data Analyses

#### Data Analyses Were Performed Using SPSS Statistics 22

In order to provide evidence for the adequacy of the psychometric proprieties for the scales used, Confirmatory Factor Analysis (CFA) was employed. For the WA measure a first-order CFA was executed, whereas for job and personal resources two second-order CFAs were performed.

Discriminant validity between the variable conceptualized as an outcome (i.e., WA) and the variables conceptualized as moderators (i.e., job and personal resources) was assessed by performing a series of CFAs aimed at testing two alternative factorial structures. The first included a single latent factor on which were loaded items from WA and from a resource conceptualized as a moderator. The second included two related factors, in which the seven items of WA were loaded on one factor and the items of a resource conceptualized as a moderator on the other factor. As a result, eighteen models were carried out, two for each resource considered as a moderator in our hypotheses.

The goodness of fit of the model was assessed with: the ratio of χ^2^ to the degrees of freedom (df), the Comparative Fit Index (CFI), the Goodness-of-fit Index (GFI), the Standardized Root Mean Square Residual (SRMR), and Root Mean Square Error of Approximation (RMSEA). According to Kline (2005), a χ^2^/df ratio of 3 or less indicates a good model fit, and less than 2 an excellent model fit. For the GFI and CFI indices, values equal to or higher than 0.90 are considered to be indicators of a satisfactory model fit (Hoyle, 1995). A value of SRMR less than 0.07 indicates good fit (Hu and Bentler, 1999). Finally, a value of RMSEA that is lower than 0.08 indicates an acceptable model fit (Byrne, 1998). In addition, for discriminant validity, the Chi-square difference test (Δχ^2^) was used to compare the alternative models.

Preliminary analyses also included means, standard deviations, Pearson’s correlations (to examine the relationship between continuous variables), and *t*-test (to examine the relationship between nominal variables and a continuous variable).

For each moderated hierarchical regression performed, predictor variables were entered into three successive steps. In the first step, the standardized indices of age and a resource was entered. In the second step, the interaction term, which is the product between the age and a resource, was incorporated in the model. In the third step, the model was adjusted for the control variables (i.e., gender, job seniority, and night shift). In cases in which the interaction term showed a significant value, the simple slope procedure recommended by Aiken and West (1991) was adopted to further examine the pattern of the relationship.

## Results

### Measurement Models

Psychometric proprieties of the measures used were found to be adequate. Regarding work ability, CFA showed an excellent fit, after adding two covariances between two couples of error terms: χ^2^ = 35.86, df = 12; χ^2^/df = 2.98; CFI = 0.92; GFI = 0.97; SRMR = 0.05; RMSEA = 0.07(0.05–0.10). The second-order CFA including all personal resources revealed an adequate structure, after incorporating four covariances between error terms (i.e., three covariances between error terms of four observed variables loaded on the resilience factor and one covariance between two error terms of two items leaded on the optimism factor): χ^2^ = 512.93, df = 267; χ^2^/df = 1.92; CFI = 0.91; GFI = 0.90; SRMR = 0.05; RMSEA = 0.05(0.05–0.06). Finally, the second-order CFA for job resources showed an excellent fit as well: χ^2^ = 344.20, df = 147; χ^2^/df = 2.34; CFI = 0.92; GFI = 0.90; SRMR = 0.05; RMSEA = 0.06(0.05–0.07).

In all the CFAs, all the observed variables significantly loaded on its corresponding latent factor and in the expected direction. Moreover, as regarded the second-order CFA, all the first-order factors positively and significantly loaded on the second-order factor.

Discriminant validity between WA and each resource of personal and job type was proved. As shown in **Table 1**, in all cases, the two-factor model, where each item leaded on its corresponding factor (**Table 1**, a column), fitted significantly better than the one-factor model, where items from work ability and a resource considered loaded on the same factor (**Table 1**, b column).

**Table 1:** Discriminant analyses between work ability and job and personal resources – Confirmatory factor Analyses: Goodness-of-fit indexes.

	One-factor model (a)						Two-factor model (b)						Model comparison (a–b)	
Resource	χ^2^(df)	χ^2^/df	CFI	GFI	SRMR	RMSEA (CI)	χ^2^(df)	χ^2^/df	CFI	GFI	SRMR	RMSEA (CI)	Δχ^2^	*p*
Decision authority (DA)	93.18 (33)	2.82	0.86	0.94	0.06	0.07 (0.06–0.09)	71.17 (32)	2.22	0.91	0.96	0.05	0.06 (0.04–0.08)	22.01	0.001
Skill discretion (SD)	291.69 (52)	5.61	0.76	0.85	0.11	0.12 (0.10–0.13)	150.47 (51)	2.95	0.90	0.93	0.07	0.07 (0.06–0.09)	141.22	0.001
Meaning of the work	198.71 (33)	6.02	0.74	0.87	0.11	0.12 (0.11–0.14)	68.78 (32)	2.15	0.94	0.96	0.05	0.06 (0.04–0.08)	129.93	0.001
(MW) Support from superior (SS)	260.13 (42)	6.19	0.81	0.85	0.13	0.12 (0.11–0.14)	97.12 (41)	2.36	0.95	0.95	0.07	0.06 (0.05–0.08)	163.01	0.001
Support from colleagues (SC)	228.81 (42)	5.44	0.78	0.87	0.11	0.12 (0.10–0.13)	89.06 (41)	2.17	0.94	0.95	0.05	0.06 (0.04–0.08)	139.75	0.001
Optimism (OPT)	234.23 (62)	3.77	0.80	0.88	0.08	0.09 (0.08–0.10)	150.20 (61)	2.46	0.90	0.93	0.07	0.06 (0.05–0.08)	84.03	0.001
Hope (HO)	257.66 (63)	4.09	0.78	0.87	0.10	0.10 (0.08–0.11)	137.98 (62)	2.22	0.92	0.94	0.07	0.06 (0.05–0.07)	119.68	0.001
Resilience (RES)	335.94 (113)	2.97	0.86	0.88	0.07	0.08 (0.07–0.09)	233.45 (112)	2.08	0.92	0.92	0.06	0.06 (0.05–0.07)	102.49	0.001
Self-efficacy (SE)	236.06 (52)	4.54	0.72	0.88	0.10	0.10 (0.09–0.12)	120.30 (51)	2.35	0.90	0.94	0.06	0.06 (0.05–0.08)	115.76	0.001

*df = degree of freedom; CFI, comparative fit index; GFI, goodness of fit index; SRMR, standardized root mean square residual; RMSEA, root mean square error of approximation.*

### Preliminary Analyses

**Table 2** shows the univariate relationships between the variables under study, including control variables. As expected, WA showed a significant and negative correlation with age. Moreover, WA was found to be positively associated with all resources considered, with the exception of support from superiors. Regarding control variables, WA was significantly and negatively associated with job seniority. Finally, *t*-test showed that those who worked on the night shift reported significantly higher levels of WA (*M* = 39.27; *SD* = 5.38) when compared with those who did not work on the night shift (*M* = 37.38; *SD* = 5.80).

**Table 2:** Means (*M*), standard deviations (*SD*), and univariate analyses (Pearson’s correlations and *t*-tests) between study variables.

	*M* (*SD*)	WA	Age	DA	SD	MW	SS	SC	OPT	HO	RES	SE	JS
Work ability (WA)	38.27 (5.67)	1											
Age	44.65 (10.02)	–0.34**	1										
Decision authority (DA)	2.85 (0.59)	0.22**	–0.12*	1									
Skill discretion (SD)	3.24 (0.59)	0.22**	–0.03	0.40**	1								
Meaning of the work (MW)	3.26 (0.62)	0.29**	–0.12*	0.26**	0.47**	1							
Support from superior (SS)	3.48 (0.86)	0.09	0.10	0.09	0.16**	0.23**	1						
Support from colleagues (SC)	2.83 (0.59)	0.23**	–0.02	0.21**	0.28**	0.31**	0.27**	1					
Optimism (OPT)	2.72 (0.44)	0.26**	–0.13*	0.07	0.16**	0.24**	0.09	0.09	1				
Hope (HO)	2.94 (0.42)	0.25**	–0.05	0.17**	0.20**	0.24**	0.03	0.03	0.47**	1			
Resilience (RES)	2.90 (0.41)	0.35**	–0.07	0.22**	0.27**	0.32**	0.05	0.17**	0.47**	0.75**	1		
Self-efficacy (SE)	2.98 (0.38)	0.21**	0.05	0.11*	0.18**	0.35**	0.16**	0.30**	0.23**	0.35**	0.40**	1	
Job seniority (JS)	10.25 (8.18)	–0.19*	0.75**	–0.00	0.14**	0.01	0.17**	0.08	–0.05	–0.00	–0.02	0.01	1
Gender (*t*-test)	–	–1.12	1.46	1.18	0.12	–1.03	–0.24	1.80	–0.31	–0.98	–0.71	0.60	2.74**
Night shift (*t*-test)	–	–2.14*	8.18**	–0.93	–0.84	0.13	0.92	0.29	0.24	0.10	–0.20	0.77	9.39**

*^∗∗^*p* ≤ 0.01 (two-tailed). ^∗^*p* ≤ 0.05 (two-tailed).*

### Moderated Regression Analyses

**Table 3** shows the results of moderated regressions assessing the moderating role of job resources in the relationship between age and WA. In the first step, in all models, *R*^2^ was significant, and age was significantly and negatively associated with WA; moreover, all the resources considered were found to be associated with WA, except for support from superiors. In the second step, the interaction effect between age and job resources was found to be significant in two of the five models carried out, suggesting that decision authority (β = 0.16, *p* = 0.003) and meaning of work (β = 0.14, *p* = 0.011) moderate the effects of age on WA. In the third step, when the models were adjusted for the control variables, both of the interaction terms kept showing significant β values (i.e., age ^∗^ decision authority: β = 0.16, *p* = 0.004; age ^∗^ meaning of the work: β = 0.14, *p* = 0.014). Regarding control variables, job seniority was found to be positively associated with WA (β = 0.22, *p* = 0.017) in one model only (JR4: support from superiors).

**Table 3:** Moderated regression analyses to assess the effect of job resources (JR) in affecting the relationship between age and work ability.

		JR 1: Decision authority			JR 2: Skill discretion			JR 3: Meaning of the work			JR 4: Support from superior			JR 5: Support from colleagues		
Step		β	*t*	*p*	β	*t*	*p*	β	*t*	*p*	β	*t*	*p*	β	*t*	*p*
1	Age	–*0.35*	–*6.25*	*0.001*	–*0.34*	–*6.14*	*0.001*	–*0.30*	–*5.42*	*0.001*	–*0.36*	–*6.42*	*0.001*	–*0.33*	–*5.94*	*0.001*
	Job resource	*0.20*	*3.57*	*0.001*	*0.20*	*3.68*	*0.001*	*0.20*	*3.58*	*0.001*	*0.11*	*1.93*	*0.055*	*0.21*	*3.85*	*0.001*
2	Age	–*0.34*	–*6.43*	*0.001*	–*0.34*	–*6.28*	*0.001*	–*0.33*	–*6.08*	*0.001*	–*0.36*	–*6.54*	*0.001*	–*0.34*	–*6.20*	*0.001*
	Job resource	*0.22*	*4.06*	*0.001*	*0.21*	*3.87*	*0.001*	*0.20*	*3.57*	*0.001*	0.09	1.76	0.079	*0.20*	*3.50*	*0.001*
	Age × job resource	*0.16*	*3.00*	*0.003*	0.08	1.51	0.131	*0.14*	*2.57*	*0.011*	0.08	1.43	0.152	0.09	1.511	0.132
3	Age	–*0.40*	–*4.99*	*0.001*	–*0.39*	–*4.64*	*0.001*	–*0.42*	–*5.05*	*0.001*	–*0.48*	–*5.71*	*0.001*	–*0.41*	–*5.07*	*0.001*
	Job resource	*0.21*	*3.86*	*0.001*	*0.19*	*3.39*	*0.001*	*0.18*	*3.29*	*0.001*	0.10	1.80	0.072	*0.19*	*3.32*	*0.001*
	Age × job resource	*0.16*	*2.89*	*0.004*	0.07	1.34	0.182	*0.14*	*2.47*	*0.014*	0.08	1.38	0.168	0.08	1.46	0.145
	Gender (1 = male)	0.03	0.51	0.608	0.03	0.48	0.626	0.03	0.53	0.595	0.03	0.49	0.626	0.05	0.93	0.356
	Job seniority	0.14	1.56	0.119	0.11	1.17	0.241	0.16	1.81	0.071	*0.22*	*2.40*	*0.017*	0.15	1.76	0.079
	Night shift (1 = yes)	0.09	1.57	0.117	0.08	1.23	0.220	0.08	1.28	0.202	0.10	1.57	0.116	0.08	1.29	0.199

1	*R^2^*	0.16^∗∗∗^			0.16^∗∗∗^			0.15^∗∗∗^			0.13^∗∗∗^			0.15^∗∗∗^		
2	*R*^2^	0.20^∗∗∗^			0.17^∗∗∗^			0.19^∗∗∗^			0.14^∗∗∗^			0.16^∗∗∗^		
3	*R*^2^	0.21^∗∗∗^			0.18^∗∗∗^			0.21^∗∗∗^			0.16^∗∗∗^			0.19^∗∗∗^		

*^∗∗∗^*p* ≤ 0.001.*

**FIGURE 1:**
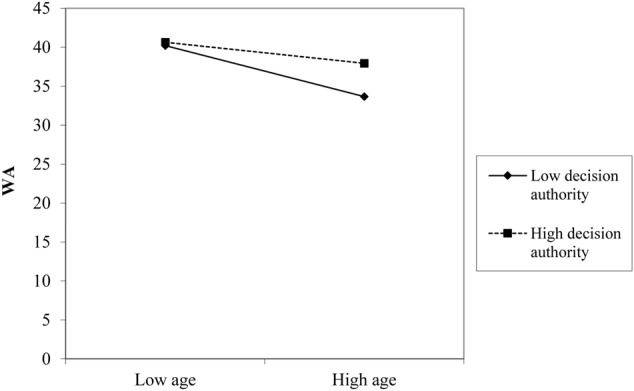
The effect of decision authority (DA) in the relationship between age and work ability (WA).

**FIGURE 2:**
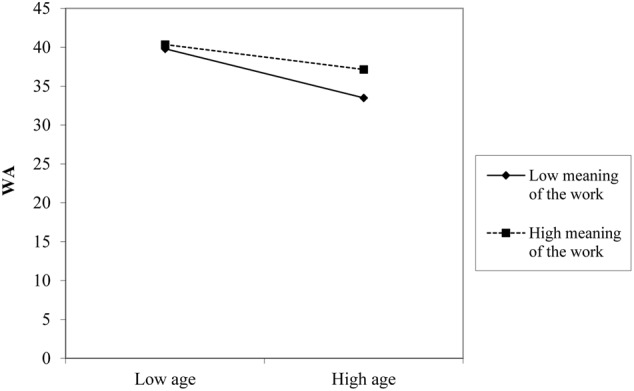
The effect of meaning of work (MW) in the relationship between age and work ability (WA).

Slope test analyses were performed in order to further examine the direction of the effects of decision authority and meaning of work in the relationship between age and WA. As regards decision authority (**Figure 1**), simple slope analysis showed that when this resource was high (+1 standard deviation, SD), the relationship between age and WA (*t* = -2.28, *p* = 0.023), albeit significant, was weaker if compared with a condition of low job resource (-1 *SD, t* = -5.93, *p* = 0.001). Similarly, (**Figure 2**), the relationship between age and WA was stronger in the condition of low meaning of work (-1 *SD, t* = -5.24, *p* = 0.001) rather than in the condition of a high meaning of work (+1 *SD, t* = -3.01, *p* = 0.03).

**Table 4** shows the moderated regressions assessing the moderating role of personal resources in the relationship between age and WA. Both in the first and in the second step, all the models reported a significant *R*^2^, age was found to be significant in all models, and all personal resources considered were found be positively associated with WA. Moreover, in two models, the interaction term was found to be significant at both steps 1 and 2, supporting the moderating role for hope (step 1: β = 0.14, *p* = 0.011; step 2: β = 0.12, *p* = 0.027) and resilience (step 1: β = 0.11, *p* = 0.022; step 2: β = 0.12, *p* = 0.029), but not for optimism and self-efficacy, in the relationship between age and WA. As regards control variables (step 3), no significant relationships with WA were reported.

**Table 4:** Moderated regression analyses to assess the effect of personal resources (PR) in affecting the relationship between age and work ability.

		PR 1: Optimism			PR 2: Hope			PR 3: Resilience			PR 4: Self-efficacy		
Step		β	*t*	*p*	β	*t*	*p*	β	*t*	*p*	β	*t*	*p*
1	Age	–*0.30*	–*5.22*	*0.001*	–*0.32*	–*5.83*	*0.001*	–*0.32*	–*5.95*	*0.001*	–*0.36*	–*6.58*	*0.001*
	Personal resource	*0.22*	*3.96*	*0.001*	*0.25*	*4.52*	*0.001*	*0.31*	*5.68*	*0.001*	*0.24*	*4.32*	*0.001*
2	Age	–*0.30*	–*5.42*	*0.001*	–*0.31*	–*5.74*	*0.001*	–*0.32*	–*6.21*	*0.001*	–*0.356*	–*6.56*	*0.001*
	Personal resource	*0.23*	*4.13*	*0.001*	*0.23*	*4.26*	*0.001*	*0.32*	*6.22*	*0.001*	*0.212*	*3.74*	*0.001*
	Age × personal resource	0.07	1.24	0.216	*0.14*	*2.56*	*0.011*	*0.12*	*2.31*	*0.022*	0.102	1.80	0.073
3	Age	–*0.30*	–*4.44*	*0.001*	–*0.39*	–*4.68*	*0.001*	–*0.40*	–*5.13*	*0.001*	–*0.382*	–*5.90*	*0.001*
	Personal resource	*0.24*	*4.12*	*0.001*	*0.22*	*4.04*	*0.001*	*0.31*	*5.95*	*0.001*	*0.208*	*3.66*	*0.001*
	Age × personal resource	0.073	1.30	0.193	*0.14*	*2.53*	*0.012*	*0.12*	*2.20*	*0.029*	0.106	1.87	0.062
	Gender (1 = male)	0.030	0.54	0.592	0.032	0.57	0.568	0.024	0.452	0.652	0.046	0.83	0.405
	Job seniority	0.076	1.23	0.221	0.135	1.49	0.138	0.135	1.623	0.106	0.097	1.62	0.107
	Night shift (1 = yes)	0.069	1.12	0.265	0.053	0.81	0.419	0.058	0.938	0.349	0.025	0.41	0.679
1		0.15^∗∗∗^			0.17^∗∗∗^			0.20^∗∗∗^			0.18^∗∗∗^		
2	*R*^2^	0.16^∗∗∗^			0.19^∗∗∗^			0.25^∗∗∗^			0.20^∗∗∗^		
3	*R*^2^	0.17^∗∗∗^			0.22^∗∗∗^			0.26^∗∗∗^			0.23^∗∗∗^		

*^∗∗∗^*p* ≤ 0.001.*

**FIGURE 3:**
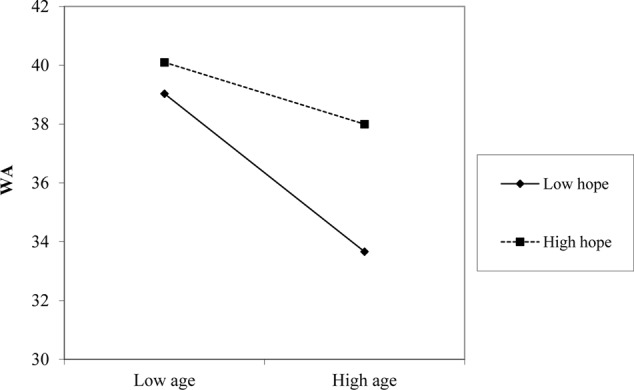
The effect of hope (HO) in the relationship between age and work ability (WA).

**FIGURE 4:**
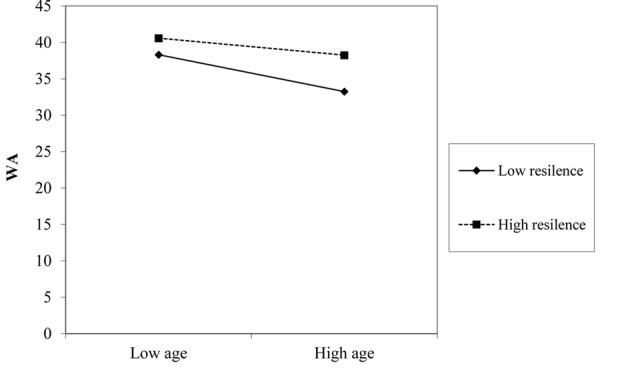
The effect of resilience (RES) in the relationship between age and work ability (WA).

Simple slope analysis (**Figures 3, 4**) showed the relationship between age and WA was stronger in conditions of low personal resources (-1 *SD*, hope: *t* = -7.34, *p* = 0.001; resilience: *t* = -5.02, *p* = 0.001) as opposed to conditions of high personal resources (+1 *SD*, hope: *t* = -1.42, *p* = 0.15; resilience: *t* = -2.11, *p* = 0.035).

## Discussion

This study had two main purposes. The first was to examine the relationship between age and WA among nurses. Regarding this point, the results confirmed H1, indicating that age is significantly and negatively associated with WA, confirming previous studies in various occupational sectors (Ilmarinen et al., 1997; Pojonen, 2001; Goedhard and Goedhard, 2005; Camerino et al., 2006; Lin et al., 2006; Monteiro et al., 2006; van den Berg et al., 2008, 2009; Godinho et al., 2016) and in the nursing profession (Camerino et al., 2006, 2008b; Golubic et al., 2009; Carel et al., 2012; Monteiro et al., 2012).

The second aim was to investigate whether job resources (decision authority, skill discretion, social support from colleagues and superiors and meaning of work) and personal resources (self-efficacy, hope, optimism, and resilience) moderate the relationship between age and WA.

All the job resources, with the exception of the support from superiors, and the four personal resources were found to be positively associated with WA. Moreover, the buffering hypothesis (H3) was partially confirmed. Two of the job resources showed a moderating effect on the relationship between age and WA. In particular, decision authority and work meaning moderate the negative effect of age on WA. Concerning personal resources, the present study confirmed that they contribute to maintaining WA (Larsson et al., 2012; Airila et al., 2014; Ng et al., 2015), specifically hope and resilience.

If health promotion and prevention represent core targets for any occupational health researcher, the considerable impact that the aging process is having on the western workforce requires more substantial efforts. In this view, these results are very important because the identification of the organizational and the personal resources that can moderate the negative effect of age on WA may support health practitioners in developing more effective and specific interventions. In this perspective, a recent campaign of the European Agency for Occupational Safety and Health (2016–2017) has been devoted to the creation of “Healthy workplaces for all ages,” stating that, due to the aging of workforce, workplace health promotion interventions cannot be postponed. As scholar and practitioners affirm, it is necessary to adopt “holistic approaches” (Brady et al., 2016) and invest in job and personal resources developing multilevel interventions focused on age-related changes (Fraccaroli et al., 2017). These interventions may include ergonomic solutions when possible, but also individual support services, which have proved to be quite useful in preventing the minor mental disorders that increase physiologically with aging (Rothermund et al., 2016), to manage crises in the transitions (i.e., retirement or changes in health status). Moreover, our study suggests that interventions that have shown a special efficacy to raise hope, resilience, and the ability to endure stress, such as mindfulness (Hülsheger et al., 2013; Malinowski et al., 2017) might be implemented.

Generally speaking, the findings from this study may contribute to human resource management, specifically in the age management perspective (Marcaletti and Garavaglia, 2014), defining human resource retention policies and practices, by emphasizing the role of job and personal resources in remedying the consequence of increasingly older workers. In particular, our findings suggest that the “importance” of a resource for a worker tends not to be absolute but relative, changing throughout the work lifespan. In this direction, the role played by the meaning of work and decision authority is very interesting and important for older workers. This confirms the literature on older workers’ motivation, which suggests that the lifespan approach should be applied to work motivation (Kanfer and Ackerman, 2004). This approach proposes that there are age-related changes in motivation toward jobs (Schalk et al., 2010) and that older workers are more motivated by the intrinsic dimensions of their job, such as the meaning of their work and their decision authority, than younger workers, who tend to be more motivated by extrinsic aspects of their job (Guglielmi et al., 2016).

This study has several limitations. The first is the cross-sectional design, as it assumes that job and personal resources are antecedents of WA; however, the opposite could also be true. For example, high levels of WA could affect the level of hope. To define the direction of the relationship, a longitudinal study design should be employed in the future. Another limitation is that all measures are self-reporting. In the future, it might be more useful to associate subjective and physical indicators to measure health and well-being at work. Finally, the healthy worker effect, which refers to the tendency of the actively employed to be in better health than the general population (Eisen et al., 2001), could act as a potential bias in the interpretation of results, as in the past, nurses with poor WA may have changed jobs or chosen early retirement.

## Author Contributions

Research conception and design/acquisition of data: DC, SV, IS, GG, and BL. Data analysis: SV, DC, IS, GG, BL, and MC. Interpretation of data: DC, IS, SV, GG, BL, and MC. Drafting the article: IS, DC, SV, GG, BL, and MC. Critical revision of the article content: DC, IS, SV, GG, BL, and MC.

## Conflict of Interest Statement

The authors declare that the research was conducted in the absence of any commercial or financial relationships that could be construed as a potential conflict of interest.

## Footnotes

1 http://www.salute.gov.it/portale/news/p3_2_1_1_1.jsp?lingua=italiano&menu=notizie&p=dalministero&id=688
